# A cyclic behavioral modeling aspect to understand the effects of vaccination and treatment on epidemic transmission dynamics

**DOI:** 10.1038/s41598-023-35188-3

**Published:** 2023-05-23

**Authors:** Abu Zobayer, Mohammad Sharif Ullah, K. M. Ariful Kabir

**Affiliations:** 1 Department of Mathematics, Bangladesh University of Engineering and Technology, Dhaka, 1000 Bangladesh; 2 Department of Mathematics, Feni University, Feni, Bangladesh

**Keywords:** Computational biology and bioinformatics, Evolution, Environmental social sciences, Mathematics and computing

## Abstract

Evolutionary epidemiological models have played an active part in analyzing various contagious diseases and intervention policies in the biological sciences. The design in this effort is the addition of compartments for treatment and vaccination, so the system is designated as susceptible, vaccinated, infected, treated, and recovered (SVITR) epidemic dynamic. The contact of a susceptible individual with a vaccinated or an infected individual makes the individual either immunized or infected. Inventively, the assumption that infected individuals enter the treatment and recover state at different rates after a time interval is also deliberated through the presence of behavioral aspects. The rate of change from susceptible to vaccinated and infected to treatment is studied in a comprehensive evolutionary game theory with a cyclic epidemic model. We theoretically investigate the cyclic SVITR epidemic model framework for disease-free and endemic equilibrium to show stable conditions. Then, the embedded vaccination and treatment strategies are present using extensive evolutionary game theory aspects among the individuals in society through a ridiculous phase diagram. Extensive numerical simulation suggests that effective vaccination and treatment may implicitly reduce the community risk of infection when reliable and cheap. The results exhibited the dilemma and benefitted situation, in which the interplay between vaccination and treatment evolution and coexistence are investigated by the indicators of social efficiency deficit and socially benefited individuals.

## Introduction

In recent years, the spread of the virus has created attention among people^1,2^, which affects people's lives, such as the covid-19 pandemic^3^, monkeypox^4^, seasonal influenza^5^, and others. Apart from country initiatives, its impact on individual or group initiatives is also noticeable^6^. Infectious diseases could be driven towards eradication if adequate and timely steps (e.g., vaccination, treatment, self-defense measure, and refinement campaign) are taken in the course of the epidemic^7^. However, many of these diseases eventually become endemic in our society due scarcity of adequate policies and timely interventions to mitigate the spread of the viruses^8,9^. Consequently, there is a need for proactive and retroactive steps toward controlling the spread of infectious diseases, particularly those for which both vaccines and treatment are available. Here, the theoretical studies of vaccination and treatment strategies have considered different effectiveness, associated costs, payoff structures, and time scales.

Based on the theory of Kermark and Mckendrick^10^, the dynamics of infectious diseases can usually be described mathematically based on compartmental models such as SIR (susceptible–infected–recovered) or SIRS models, with each term referring to a “compartment” in which an individual can reside. Recently, Covid-19 inclined the attention of mathematicians, and they have tried to inflict an approximate solution by bearing different models^11^. To understand the mechanism of infectious disease transmission, several authors have studied various kinds of epidemic models by considering other compartment models such as SI^12^, SIS^13^, SIR^14–20^, SIRS^21^, SEIR^22,23^, SVEIR^24^, and many more. Mathematical modeling has been successfully used in constructing control strategies with suitable interventions for various diseases, such as vaccination, treatment, and quarantine^25–31^. Variations of standard, SIR, SIRS, and SEIR epidemiological models are considered to determine the sensitivity of these models to different parameter values that may not be fully known when the models are used to investigate emerging diseases^32,33^. Previous works above explored that vaccination, quarantine, and treatment would retrench contagious disease in a simple dynamic aspect on local time scales. The current study aims to develop a theoretical epidemic model embedding both vaccination and treatment as a cyclic model.

An extensive evolutionary game theory study studied the influence of people's vaccination decisions on imposed control policies, reducing the epidemic's spreading severity^34–38^. Prior research has shown that a game-based approach to epidemiological vaccination may accurately forecast infection risk in vaccinated and unvaccinated people^39,40^. Based on the vaccine performance and reliability, Kuga et al.^41^ explained the framework of the vaccination effectiveness and efficiency model from the standpoint of the vaccination game. To control disease, the vaccine properties, people's judgment, social networks, and neighbors' choices influence how people make apposite decisions. As a result, it is crucial to examine how different elements affect both vaccine and treatment acceptance^42–48^. Here, we use the SVITR-type epidemic model to thoroughly analyze the combined impact of these two types of protective measures: proactive and retroactive. In this situation, the vaccine's efficiency and treatment duration serve as a control parameters. Individuals' ability to update their methods by emulating those who appear to have adopted more effective techniques must be integrated into the model^49,50^. To characterize this process formally, we need to build a model that incorporates mathematical epidemiological with game-theoretic dynamics. For example, Bauch et al.^51^ made and tested a model that blends epidemiological dynamics with replicator dynamics from evolutionary game theory to describe individuals' copying behavior during disease outbreaks. However, people's attitudes toward vaccination and treatment reflect their inherent recognition to choose between vaccine acceptance and risk of infection. This approach incorporates two types of game aspects (proactive and retroactive) on a local time scale^52^ to illuminate the framework of cyclic disease dynamics embedded with vaccination and treatment provision^51,52^.

This work introduces a new indicator, *“socially benefited individual,”* termed SBI for vaccination and treatment provisions. This approach benefits individuals from society who get advantages from participating in the vaccine program or treatment. Besides, we also explore the idea of a dual dilemma by considering the *“social efficiency deficit”* (SED)^53,54^ that presents the roles of vaccination (before infection) and treatment (after infection) games. Both evolutionary games occur on a local time scale (single season) that is affected by various factors concerning vaccine efficacy, vaccine cost, treatment cost, and treated time (facilities). We impose a pre-emptive intervention policy that controls disease before the infection spreads at an early stage that depends on the individual’s choice. On the other hand, the treatment strategy can be considered a “let-down” intervention in which people will recover faster. Utilizing our new idea assisted by the evolutionary game theory approach on epidemiology for vaccination and treatment game model, we explore the impact of the dual-dilemma and social benefit situations by presenting line graphs and phase diagrams. Such a social benefit approach and dual-dilemma situation in the same framework, perhaps quite omnipresent in the real world, has not been studied in related earlier works.

## Model and method

**Figure 1:**
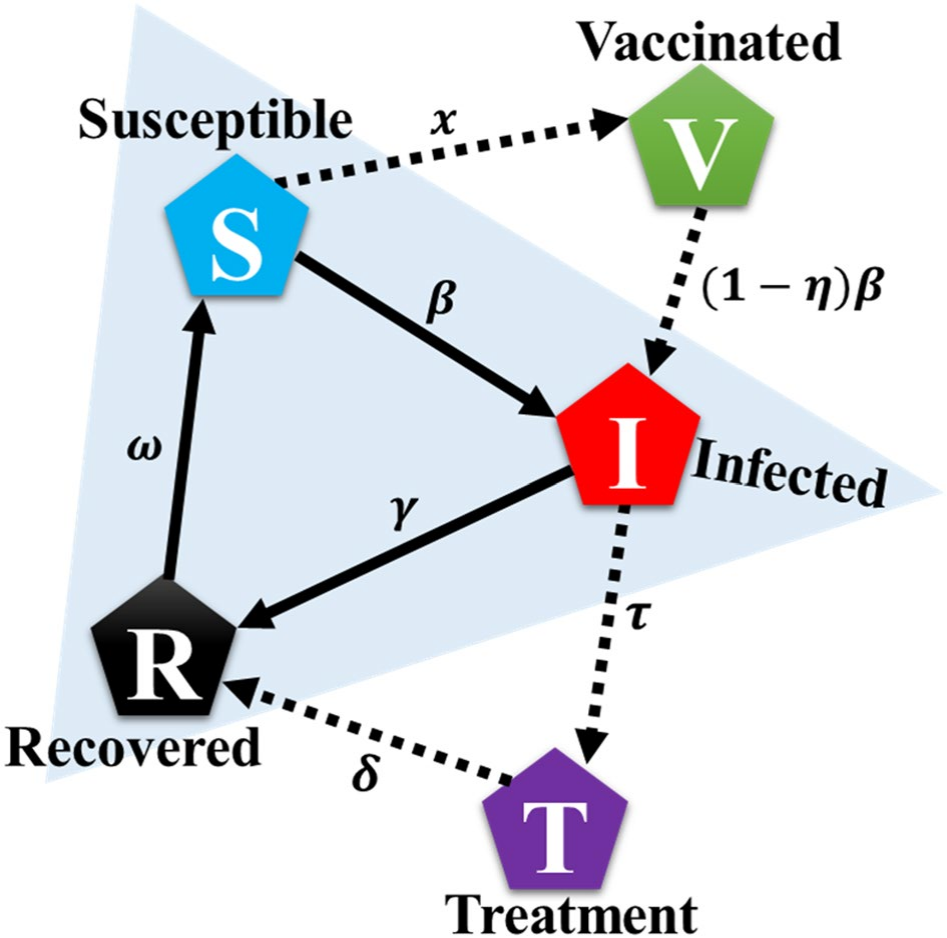
The five Compartment are susceptible ($S$), infected ($I$), vaccination ($V$), treatment ($T$), recovery ($R$). Susceptible people can be infected if they do not get the vaccine. Infected person can recover after taking treatment, also without treatment can gain recovery. After recovering, the person may be susceptible again. The exhaustive cycle is a continuous process. Susceptible people can be infected by infection rate $\beta$ and they (individuals) can get vaccinated at rate $x$. The vaccinated individuals infected at the rate $(1-\eta )\beta$ if the vaccine is not perfectly effective. After infected, two different cases can be happened to an infected person; either individuals will get treatment at $\tau$ rate or they will recover naturally by recover rate, $\gamma$. Individuals can go recover state at rate $\delta$ termed as treated recovery rate. Finally, recovered, or immunized people will susceptible again after losing immunity at a rate $\omega$.

This section presents the dynamic model for the disease spreading and the embedded vaccine and treatment behavioral dynamics among the individuals in society. We assume SVITRS cyclic epidemic model, in which the total population is divided into five epidemiological compartments: susceptible $(\mathrm{S})$, vaccinated $(V)$, infected $(\mathrm{I})$, treatment $(T)$, and recovered (*R*) individuals (Fig. 1). Here, we use two control measures, namely, vaccination and treatment, to control more optimally the spread of infection from a community because sometimes only one control variable may be challenging to eradicate the disease successfully. A susceptible individual becomes infected at a disease transmission $\beta$ and alternatively can participate vaccine program at a dynamic rate $x$. A vaccinated may be infected at the rate $(1-\eta )\beta$, called vaccine efficiency aspect, even after participating in the vaccine program, where $\eta$ denotes vaccine effectiveness $(0\le \eta \le 1)$. After infection, two different cases can happen to an infected person. Either individual will get treatment at rate, $\tau$ or they will recover naturally at rate $\gamma$. A treated individual recovers after treatment at a rate $\delta$. Finally, an individual who has recovered after infection is immune; they can get susceptible again at a waning immunity rate $w$. The dynamical equation of SVITRS is given by,

1 $$\dot{S}=-\beta SI-xS+\omega R,$$

2 $$\dot{V}=xS-\left(1-\eta \right)\beta VI,$$

3 $$\dot{I}=\beta SI+\left(1-\eta \right)\beta VI-\gamma I-\tau I,$$

4 $$\dot{T}=\tau I-\delta T,$$

5 $$\dot{R}=\delta T+\gamma I-\omega R.$$

Here, $S\left(t\right)+V\left(t\right)+I\left(t\right)+T\left(t\right)+R\left(t\right)=1$. To solve the above sets of differential Eqs. (1,2,3,4,5) against time numerically, we consider explicit finite difference method with the initial values as, $S(0)\approx 1.0$,$V\left(0\right)\approx 0.0$, $I(0)\approx 0.0$, $T\left(0\right)=0.0$, and $R\left(0\right)=0.0$. We provide detailed theoretical analysis in the supplementary Information text^55–71^.

### Behavioral dynamics

Individuals can take vaccination and treatment based on their interests and their strategy by observing how many people are infected at a given time in a single season. In the behavioral dynamics, each participant can choose proactive intervention whether to participate vaccine program or not, depending on the vaccine cost and associated factors. Further, if infected individuals want to take treatment facilities termed retroactive provision, they compare their strategies to recover time, treatment cost, and disease incidence. Thus, if the individual becomes vaccinated and treated at a vaccination rate $(x)$ and a treatment rate $(\tau )$, the equation that describes the human behavioral dynamics are^49–51^,

6 $$\dot{x}=mx\left(1-x\right)\left[-{C}_{v}V+{C}_{i}I\right].$$

7 $$\dot{\tau } = m\tau \left( {1 - \tau } \right)\left[ { - C_{t} T + C_{i} I + \omega \left( {\frac{1}{\gamma } - \frac{1}{\delta }} \right)} \right].$$

Here, ${C}_{v}, {C}_{t},$ and ${C}_{i}$ represent the cost of vaccination, treatment, and disease, respectively. The circumstances an individual encounters during an epidemic season quantify the costs they bear and influence their ultimate social payoff, determining whether they experience gains or losses. The relative cost of vaccination $({C}_{V})$ and treatment $({C}_{T})$ as compared with the disease cost is employed here as a determining factor to determine the payoff for individuals, ${C}_{V}={C}_{v}/{C}_{i}$, ${C}_{T}={C}_{t}/{C}_{i}$ and $W=w/{C}_{i}$, where, $0\le {C}_{V}\le 1, 0\le {C}_{T}\le 1, 0\le w\le 1$ and ${C}_{i} (=1)$. Then, from Eqs. (6) and (7),

8 $$\dot{x}=mx\left(1-x\right)\left[-{C}_{V}V+I\right].$$

9 $$\dot{\tau } = m\tau \left( {1 - \tau } \right)\left[ { - C_{T} T + I + W\left( {\frac{1}{\gamma } - \frac{1}{\delta }} \right)} \right].$$

The term in Eq. (8), $\left[-{C}_{V}V+I\right]$, is designed to present the risk trade-off for an individual between cooperation and defection, and its sign (positive or negative) decides whether to vaccinate. When the cost of vaccination rises, the corresponding term $(-{C}_{V}V)$ becomes negatively impacted. Additionally, if a privileged group of individuals chooses not to vaccinate, it would have a negative impact, resulting in free-riding and hindering the possibility of achieving herd immunity. On the other hand, if the risk of infection for individuals increases due to factors such as a high fraction of infected individuals, the term would be positive, leading to an increase in the vaccination rate.

Similarly, in Eq. (9), the term $\left[-{C}_{T}T+I+W\left(\frac{1}{\gamma }-\frac{1}{\delta }\right)\right]$ is the payoff gain for treatment strategies potentially indicates how much more beneficial instead taking treatment (treated) is than not taking treatment (untreated) is in terms of disease duration. With increase of the accelerated recovery rate $\delta$ as compared with natural recover rate $\gamma$, the treatment provides a patience an immediate recovery from illness. In details, ${\delta }^{-1}$ represents the average number of days for recovery under treatment, in contrast to ${\gamma }^{-1}$ which represents the average number of days for unaided recovery. If ${\gamma }^{-1}-{\delta }^{-1}\gg 0$, indicates that the utility of treatment is significantly high, then opting for treatment should be prioritized, and a positive sign decides to incline to treatment. Thus, $\left(\frac{1}{\gamma }-\frac{1}{\delta }\right)$ implies ‘willingness’ amid infected people to take the treatment than doing nothing. Finally, it is important to highlight that *m* is incorporated as a proportional constant that accounts for the inertial effect of the social dynamic being considered and $\omega (=1)$ indicates the proportional constant that converts time duration to the relative cost of recovery duration.

### Average social payoff

To establish the average social payoff (ASP) at the end of the epidemic season, we consider the combined effect of vaccination and treatment in the same context. The ASP for the evolutionary game theory aspect at Nash equilibrium is given by,

10 $${ASP}^{NE}=-{C}_{V}V\left(\infty \right)-{C}_{T}{\int }_{0}^{\infty }T\left(\theta \right)\,d\theta -{\int }_{0}^{\infty }I\left(\theta \right)\,d\theta .$$

where ${ASP}^{NE}$ indicates the payoff at Nash equilibrium (NE), estimated when both games (vaccination and treatment) have arrived at a steady state on the local time scales.

### Social efficiency deficit (SED)

Social efficiency deficit (SED) defines as the difference between the result of the evolutionary train (which can be evaluated by the Nash equilibrium NE) and the optimum solution (without EGT). The payoff at the NE is obtained by taking an evolutionary game presence, whereas the optimal social gain is remarkable in a model of any complicacy. Therefore, one can evaluate the SED in any context and forecast the phenomenon of a social dilemma; if the SED is positive, the gap exists; if it is zero, the evolutionary train matches the optimum ($SED=0$ implies no social dilemma). The SED is given by,

11 $$SED={ASP}^{SO}-{ASP}^{NE}.$$

where ${ASP}^{SO}$ and ${ASP}^{NE}$ define the payoff at the optimal social situation and Nash equilibrium, respectively.

Here, we introduce SED that explicitly reveals the underlying social dilemmas in the vaccination and treatment game. The social dilemma exists under certain combinations of the model parameter, such as vaccine efficacy, treatment duration and their associated cost. Now, we consider the combined impact of vaccination and treatment in the same context.

In according to the abovementioned conceptual definition, SED in the current model for both vaccination and treatment games are given by,

12 $${SED}_{V}={ASP}_{{x}_{k}}^{{X}^{OS}}-{ASP}^{NE},$$

13 $${SED}_{T}={ASP}_{ {\tau }_{k}}^{{T}^{OS}}-{ASP}^{NE}.$$

The ${ASP}_{{x}_{k}}^{{X}^{OS}}$ and ${ASP}_{ {\tau }_{k}}^{{T}^{OS}}$ are the maximum quantity of average social payoff defined as the optimal social situation of vaccination and treatment. The superscript ‘OS’ indicates the optimal social situation of the respective strategy. In-depth, the terms ${ASP}_{{x}_{k}}^{{X}^{OS}}$ and ${ASP}_{ {\tau }_{k}}^{{T}^{OS}}$ reflect the statement that the maximum ASP is obtained for varying *x* ranging from 0 to 1 (treatment strategy on EGT) and varying $\tau$ from 0 to 1(vaccination strategy on EGT), respectively. It can also be noted that when $x$ (or $\tau$) varies with a constant ranging from 0 to 1 (without EGT), the alternate strategy $\tau$ (or $x$) follows the EGT approach. On the other hand, ${ASP}^{NE}$ indicates the average social payoff at the NE, estimated when both games, vaccination and treatment have occurred together on the framework of EGT.

### Social benefitted individuals (SBI)

To introduce the concept of socially benefitted individuals (SBI) on an epidemic model embedding with EGT, we consider the fraction of individuals who benefit from either vaccination or treatment. For SBI, we first calculate the final epidemic size (FES) in the absence of vaccination and treatment, FESNV and FESNT, respectively, at equilibrium. Next, vaccination and treatment game strategy are calculated at NE in the presence of either vaccination or treatment strategies, defined by FESV and FEST. Finally, we formulate the SBI as follows,

14 $${SBI}_{V}\left(\infty \right)={\mathrm{FES}}_{NV}\left(\infty \right)-{\mathrm{FES}}_{V}\left(\infty \right),$$

15 $${SBI}_{T}\left(\infty \right)={\mathrm{FES}}_{NT}\left(\infty \right)-{\mathrm{FES}}_{T}\left(\infty \right).$$

We consider the explicit finite difference method to solve the proposed epidemic model that belongs to the sets of differential equations for a single season numerically. Here, we presume the initial values as, $S(0)\approx 1.0$, $V\left(0\right)=0.0$, $I(0)\approx 0.0$, $T\left(0\right)=0.0$, and $R\left(0\right)=0.0$. Throughout the time step is to consider $\Delta t=1$, meaning both strategy and epidemic dynamics update daily (per day).

## Result and discussion

In this section, we numerically explore the SVITR model; the results are presented for the line graphs and 2D phase diagrams in the aspect of the evolutionary game theory and cyclic epidemic model. The convene impact of proactive vaccination and the retroactive treatment policy based on human behavior depends on the vaccination cost, treatment cost, and corresponding factors. We extensively analyzed the vaccination and treatment cost, vaccination effectiveness $(\eta )$, and recovery rate, considering the other sensible parameters. We first pursued the line graphs of infected individuals without the game and with game cases for varying control parameters. In the second case, we present the two-dimensional phase diagram of final epidemic size (FES), vaccination coverage (VC), the fraction of treated people (FTR), and average social payoff (ASP) as a measure of policy burden to society while varying two parameters. We also introduce the SED (social efficiency deficit), the expresses the radical social dilemmas in the vaccination and treatment games.

First, we briefly analyze the current model and its solution theoretically for the only epidemic model without using evolutionary game theory (EGT) (see Appendix). The model and its solution are positive and bounded for the finite population N(t)^66^. We explored the existence of a uniformly stable solution for the model using the well-known Lipchitwz stability theorem. We examined the model's reproduction number (basic and effective), local and global stability, and strength number to analyze stability conditions and wave properties. Finally, the Lyapunov function backed by second derivatives were also studied, providing information regarding the tendency toward curvature.

**Figure 2:**
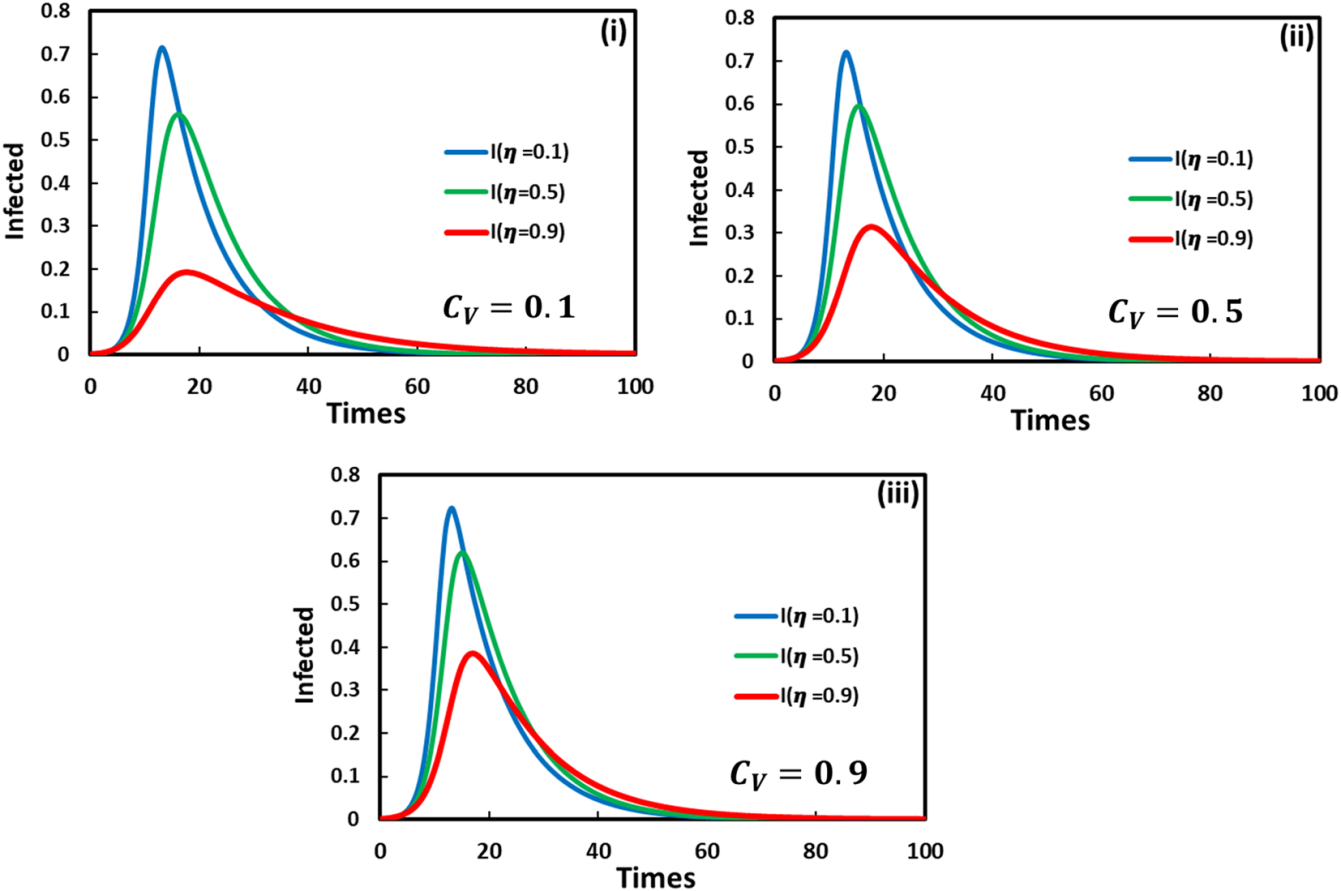
Vaccinated game and without treatment game. The time sequence of the infected individuals is presented for varying vaccine efficacy $\eta =0.1, 0.5$ and $0.9$ in each panel colored with blue, green, and red, respectively. Here panel (i), (ii), and (iii) display for vaccine cost ${C}_{V}=0.1, 0.5$ and $0.9$. Other parameters are, $\beta =0.8333$, $\gamma =0.333,$ and $\omega =0.01$.

Figure 2 shows the interplay between vaccination cost and vaccine efficiency when the treatment strategy is inactive. In each panel, the fraction of infected individuals will be minimal when vaccine effectiveness is higher indicating that higher reliability of vaccine attracts individuals to participate in vaccine programs in reducing disease. So, the primary benefit of vaccine efficiency is to reduce infection rates. However, vaccine efficiency depends on other factors, such as vaccination cost. While vaccination cost increases, at the same time, the ability to buy vaccines is decreased, and consequently, the infection rate increases. If a vaccine is cheap, the population responds in a couple of manners whereby everyone vaccinates or not. To compare panel (i) and panel (ii) for vaccine cost ${C}_{V}=0.1$ and ${C}_{V}=0.9$, if the vaccination cost is low, then the infection is reduced.

**Figure 3:**
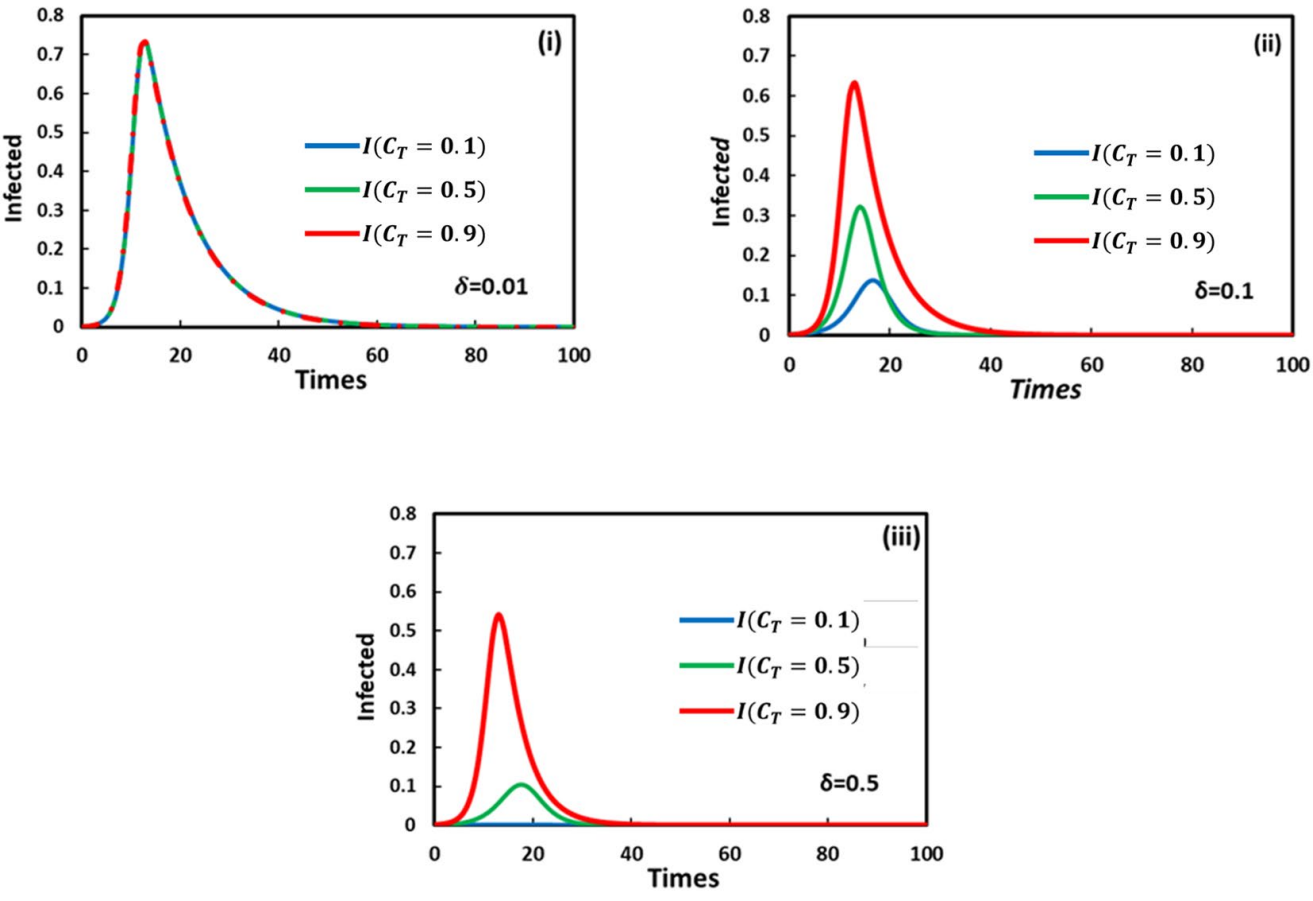
Treatment game and without vaccination game. The time sequence of the infected individuals is presented for varying treatment cost, ${C}_{T}=0.1, 0.5$ and $0.9$ in each panel colored with blue, green, and red, respectively. Here panel (i), (ii), and (iii) display for vaccine cost $\delta =0.01, 0.1$ and $0.5$. Other parameters are, $\beta =0.8333$, $\gamma =0.333,$ and $\omega =0.01$.

To examine the treatment effect on the epidemic (without vaccination), we represent Fig. 3. Here, the impact of the natural and treated recovery rates is considered to show the human behavior on treatment services or hospital facilities against a particular disease. The treatment strategy is not working when the treated recovery rate $(\delta )$ is less than the natural recovery rate $(\gamma )$ (panel (i)). As a result, maximum infections spread because of a lack of proper treatment (three lines coexist). Besides, on higher treatment recovery than natural recovery rate, $\delta >\gamma$, whenever treatment cost increases in such a periodic time, individuals lose their hope to take treatment (panel (ii) and (iii)). The most expensive treatment costs are avoided irrespective of recovery rate, and eventually, the fraction of infections during the epidemic season is maximized. Thus, it’s clear that if the treated recovery rate is faster and treatment costs are lower, the number of infected individuals will be lower.

**Figure 4:**
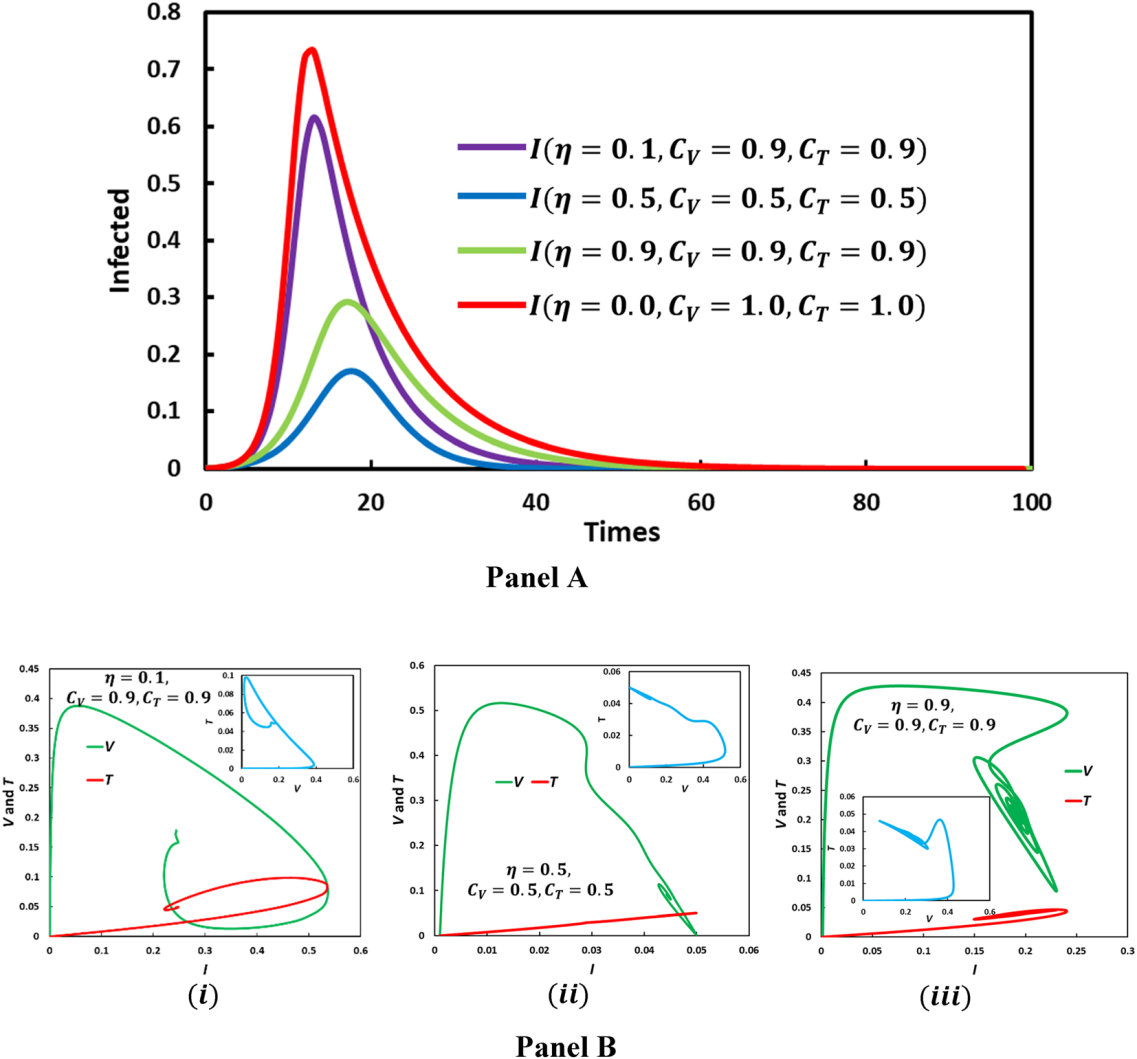
Vaccinated and Treatment game. (**A**) The time sequence of the infected individuals is presented for varying vaccine efficacy, vaccine cost and treatment cost as $(\eta , {C}_{V},{C}_{T})=(0.1, 0.9, 0.9)$, $(0.5, 0.5, 0.5)$, $(0.9, 0.9, 0.9)$ and $(0.0, 1.0, 1.0)$ colored with purple, blue, green, and red, respectively. (**B**) Phase trajectories projected on infected versus vaccinated and treatment individuals (I-V colored green and I-T colored red) under the influence of evolutionary game theory on the time scale. Also, the blue-colored trajectory displays on the inset for the coexistence of vaccination versus treatment individuals. The sub-panels (i), (ii), and (iii) present for $(\eta , {C}_{V},{C}_{T})= (0.1, \mathrm{0.9,0.9}), (0.5, \mathrm{0.5,0.5})$ and $, (0.9, 0.9, 0.9)$. Other parameters are, $\beta =0.8333$, $\gamma =0.333, \gamma <\delta$, and $\omega =0.01$.

In Fig. 4A,B, both vaccination and treatment strategies are considered to show the combined impact of intervention policies on the epidemic. In Panel A, the infected individuals depend on vaccine efficiency, vaccination, and treatment costs. When the vaccination and treatment are cheap, individuals can take more vaccines and get treatment, but the infection rate remains down. The reduced tendency of infected individuals controls remarkably when the vaccine reliability increases (higher effectiveness). The most expensive rate of vaccines is rejected even though they are very productive. Curiously, cheaper vaccines may achieve less coverage with increased efficiency, but this is because they are better at controlling outbreaks. As opposed, treatment increases vaccination coverage when the vaccine is rightly priced and sufficiently efficacious.

To investigate the coexistence of vaccination and treatment, we explore the trajectories of vaccination and treatment against infected individuals, and these are drawn in panel B, Fig. 4 for three cases, namely $(\eta , {C}_{V},{C}_{T})=$ (i) $(0.1, \mathrm{0.9,0.9}),$ (ii) $(0.5, \mathrm{0.5,0.5})$ and (iii) $(0.9, 0.9, 0.9)$. Panel B displays that all trajectories starting from zero approaches an equilibrium point after completing loops. As a general inclination, we could observe the interplay between vaccination and treatment strategies when infected individuals change and vaccinated show a higher fraction of the population with increasing vaccine efficacy rate eta. The mechanism behind this is that the vaccinated state dominates with increasing vaccine efficacy, while the treatment strategy dominates with lower vaccine efficacy. Panel B also presents the trajectory of the interplay between vaccination and treatment on the inset (blue-colored trajectory); the disease is somehow controlled by suitable provisions from either vaccination or treatment.

**Figure 5:**
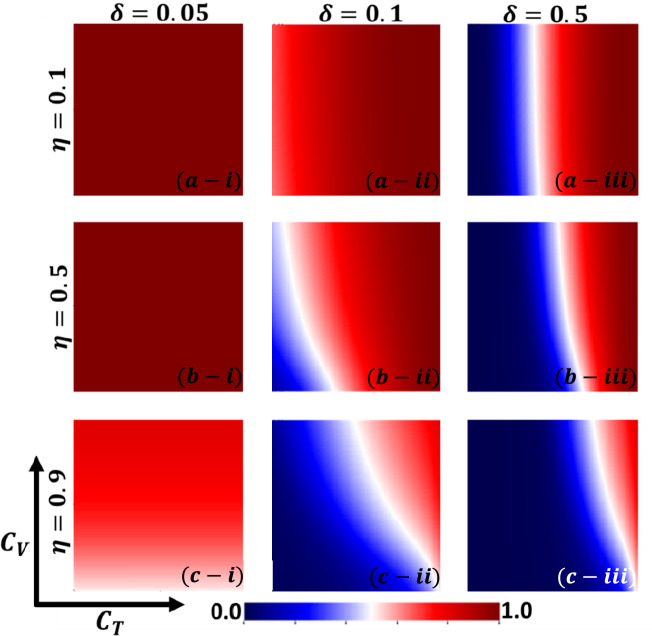
The 2D heatmap of the final epidemic size (FES) is present by varying two parameters: the *x*-axis contains treatment cost (${C}_{T}$) and the *y*-axis is vaccination cost (${C}_{V}$). In this figure, the first, second, and third rows display the result of varying the vaccine efficiency (a-*) $\eta =0.1$, (b-*) $\eta =0.5$, and (c-*) $\eta =0.9$. Also, the first, second and third columns show the result of varying the treatment duration rate: (*-i) $\delta =0.05$, (*-ii) $\delta =0.1$, and (*-iii) $\delta =0.5$. Other parameters are, $\beta =0.8333$, $\gamma =0.333$, and $\omega =0.01$.

**Figure 6:**
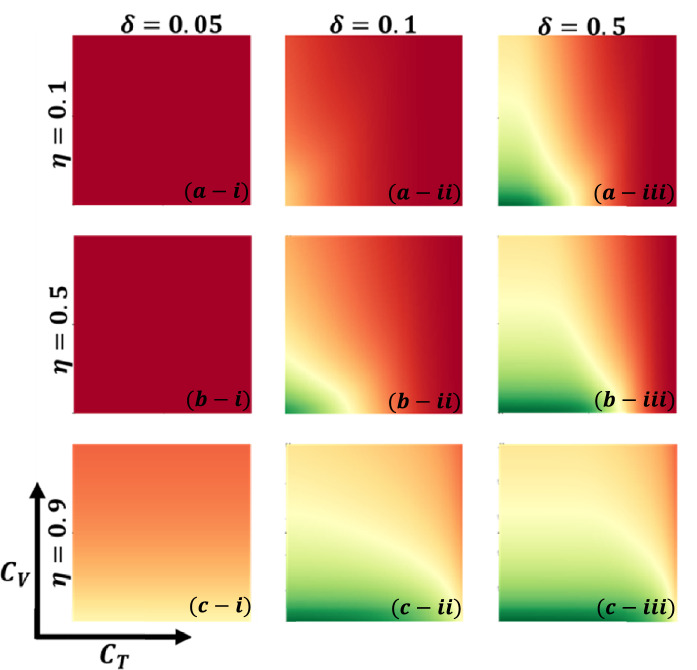
The 2D heatmap of the vaccination coverage (VC) is present by varying two parameters: the *x*-axis contains treatment cost (${C}_{T}$) and the *y*-axis is vaccination cost (${C}_{V}$). In this figure, the first, second, and third rows display the result of varying the vaccine efficiency (a-*) $\eta =0.1$, (b-*) $\eta =0.5$, and (c-*) $\eta =0.9$. Also, the first, second and third columns show the result of varying the treatment duration rate: (*-i) $\delta =0.05$, (*-ii) $\delta =0.1$, and (*-iii) $\delta =0.5$. Other parameters are, $\beta =0.8333$, $\gamma =0.333$, and $\omega =0.01$.

**Figure 7:**
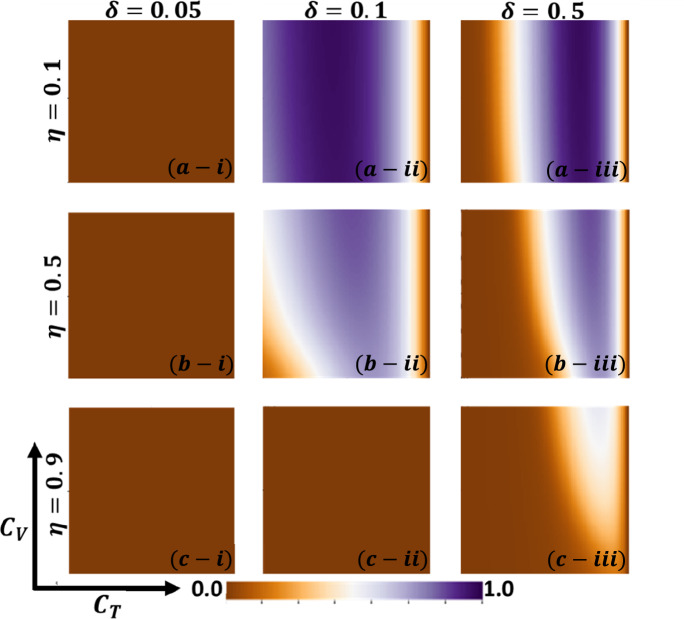
The 2D heatmap of the fraction of treated individuals (FTR) is present by varying two parameters: the *x*-axis contains treatment cost (${C}_{T}$) and the *y*-axis is vaccination cost (${C}_{V}$). In this figure, the first, second, and third rows display the result of varying the vaccine efficiency (a-*) $\eta =0.1$, (b-*) $\eta =0.5$, and (c-*) $\eta =0.9$. Also, the first, second and third columns show the result of varying the treatment duration rate: (*-i) $\delta =0.05$, (*-ii) $\delta =0.1$, and (*-iii) $\delta =0.5$. Other parameters are, $\beta =0.8333$, $\gamma =0.333$, and $\omega =0.01$.

**Figure 8:**
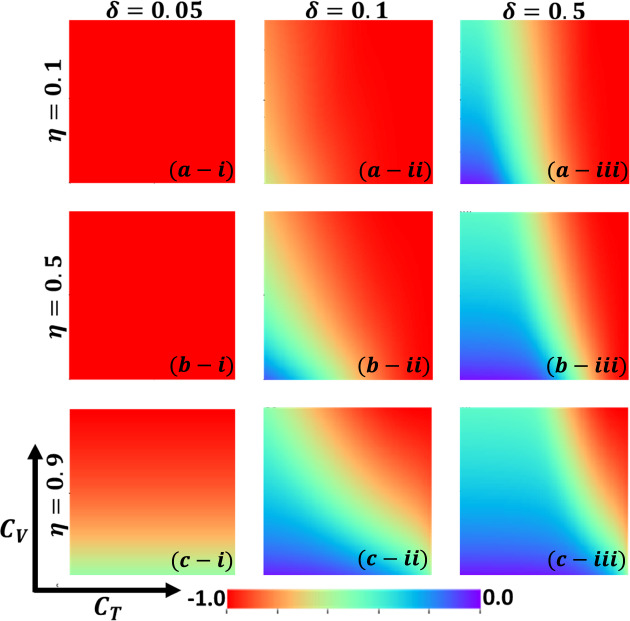
The 2D heatmap of the average social payoff (ASP) is present by varying two parameters: the *x*-axis contains treatment cost (${C}_{T}$) and the *y*-axis is vaccination cost (${C}_{V}$). In this figure, the first, second, and third rows display the result of varying the vaccine efficiency (a-*) $\eta =0.1$, (b-*) $\eta =0.5$, and (c-*) $\eta =0.9$. Also, the first, second and third columns show the result of varying the treatment duration rate: (*-i) $\delta =0.05$, (*-ii) $\delta =0.1$, and (*-iii) $\delta =0.5$. Other parameters are, $\beta =0.8333$, $\gamma =0.333$, and $\omega =0.01$.

Aside from the line graph along the time step, we now draw another set of results as a phase diagram in Figs. 5, 6,7, and 8 at the equilibrium point, expressed by the parameters of treatment cost $({C}_{\mathrm{T}})$ and vaccination cost $({C}_{\mathrm{V}})$ that describes the underlying social dilemmas in the vaccination and treatment game. Figures 5, 6, 7, and 8 display the final epidemic size (FES), vaccination coverage (VC), treated individuals (TR), and average social payoff (ASP). Throughout, the first, second, and third columns show the result of varying the vaccine efficiency, $\eta =0.1$, $\eta =0.5$, and $\eta =0.9$ in panels (*-i), (*-ii), and (*-iii), respectively. On the other hand, the first, second, and third rows display the result of varying the treated recovery rate, $\delta =0.05$, $\delta =0.1$, and $\delta =0.5$ depicted in panels (a-*), (b-*) and (c-*), respectively. the higher final epidemic size (FES) was observed for the lower treated recovery rate $(\delta =0.05)$ and less vaccine efficacy ($\eta =0.1$). Only reduced FES is obtained for high vaccine efficacy ($\eta =0.9$); people are taking a vaccine for low vaccine cost and reduced infection. Decreasing the vaccination cost at higher vaccine efficacy with treated period remains constant; it occurs less infection, which means individuals encourage to take vaccination and infection level become less. Thus, if the treatment is not favorable for individuals ($\delta <\gamma$), people only participate vaccine program (Fig. 6c-i) and avoid taking treatment (Fig. 7c-i). Next, for the higher medical (treatment) facilities that accelerate recovery (delta greater than gamma) duration, lower treatment and vaccination cost reduce the infection level, remarkably (panel (*-ii) and (*-iii)). Lower treatment costs with higher vaccine costs, individuals take treatment and are less infected (Figs. 5, 6, and 7). On the other hand, with higher treatment costs for lower vaccine costs and higher vaccine reliability, individuals take vaccines and avoid infection. In panels (c-ii) and (c-iii), for maximum vaccine efficiency, most individuals take the vaccine to avoid infection when the low vaccination cost, but higher treatment cost arises. Therefore, VC (vaccination coverage) depends on the increase or decrease of vaccine efficiency and treatment duration. However, increasing the vaccination cost and reducing the treatment cost attracted the individual to the treatment, which enhanced the FTR when the treatment duration was high. In addition, the higher vaccine efficiency and treatment recovery rate attract individuals to the treatment strategy. In contrast, a lower vaccine efficiency and recovery speed hamper the treatment-seeking behavior (lower FTR).

Now, by comparing Figs. 6 and 7, the results show some interesting phenomena when the treatment duration is greater than the natural recovery rate and the vaccine efficacy of higher. Although it’s expected that lower treatment cost attracts people to treatment in hospitals or clinics, our results show a distinct tendency when both vaccine and treatment are considered. If vaccine reliability is higher, irrespective of lower treatment cost or highly facilitated treatment, people are prone to take vaccines rather than be treated or infected. Finally, Fig. 8 represents the phase diagram of average social payoff (ASP) while varying the vaccination and the treatment cost with vaccine efficiency and treatment duration. It observed that lower vaccine and treatment cost brings higher ASP, meaning society reaches its optimal position when vaccine efficacy is higher, and $\delta >\gamma$.

**Figure 9:**
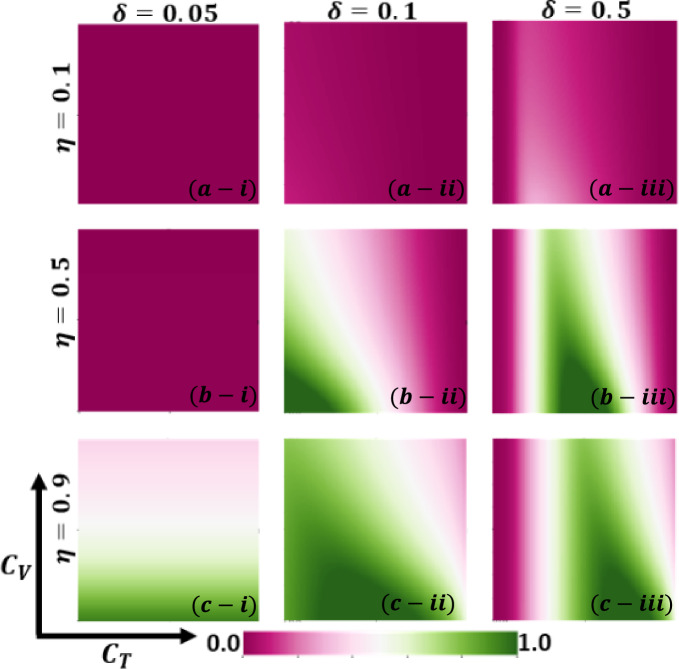
The 2D heatmap of the socially benefitted individuals from vaccination (${\mathrm{SBI}}_{\mathrm{V}}$) is present by varying two parameters: the *x*-axis contains treatment cost (${C}_{T}$) and the *y*-axis is vaccination cost (${C}_{V}$). In this figure, the first, second, and third rows display the result of varying the vaccine efficiency (a-*) $\eta =0.1$, (b-*) $\eta =0.5$, and (c-*) $\eta =0.9$. Also, the first, second and third columns show the result of varying the treatment duration rate: (*-i) $\delta =0.05$, (*-ii) $\delta =0.1$, and (*-iii) $\delta =0.5$. Other parameters are, $\beta =0.8333$, $\gamma =0.333$, and $\omega =0.01$.

**Figure 10:**
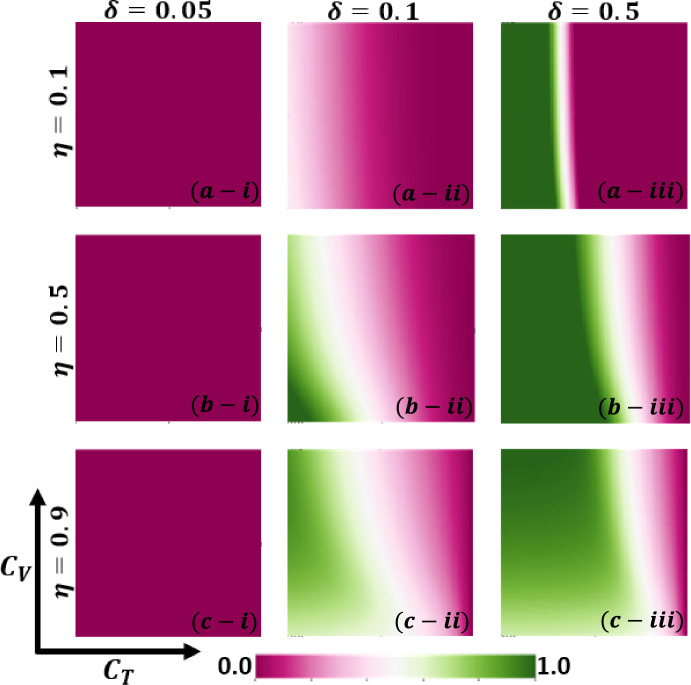
The 2D heatmap of the socially benefitted individuals from treatment (${\mathrm{SBI}}_{\mathrm{T}}$) is present by varying two parameters: the *x*-axis contains treatment cost (${C}_{T}$) and the *y*-axis is vaccination cost (${C}_{V}$). In this figure, the first, second, and third rows display the result of varying the vaccine efficiency (a-*) $\eta =0.1$, (b-*) $\eta =0.5$, and (c-*) $\eta =0.9$. Also, the first, second and third columns show the result of varying the treatment duration rate: (*-i) $\delta =0.05$, (*-ii) $\delta =0.1$, and (*-iii) $\delta =0.5$. Other parameters are, $\beta =0.8333$, $\gamma =0.333$, and $\omega =0.01$.

To realize how different parameter settings affect the benefitted individuals from intervention games: vaccination, or treatment, we interpreted socially benefitted individuals ${SBI}_{V}$ (vaccination) and ${SBI}_{T}$ (treatment) in Figs. 9 and 10, respectively. Here, we adopt a new indicator termed socially benefitted individuals (SBI), referring to Eqs. (14) and (15) from the fraction of the population gap between FES of control strategies and without controls at equilibrium. In Fig. 9, we can observe that individuals participating in vaccine programs can get more advantages from vaccination when efficacy is higher and vaccine cost is minimum. However, irrespective of the lower cost of vaccination, if the treatment cost is low, people benefit less (panels b-iii and c-iii). In Fig. 10, we can see that there are no treated benefited people for treatment (${SBI}_{T}$) when $\delta <\gamma$; treatment duration (treatment to recover) is higher than natural recovery (see panel (*-i)). These phenomena indicate that if the medical/clinic/drug facilities need to be better equipped and work effectively to recover from disease faster, people are not getting social benefits from treatment. However, for $\delta >\gamma$, individuals benefit from treatment when treatment costs are lower. Interestingly, increases in vaccine efficacy somehow increase the fraction of aided people. But for higher vaccine efficacy ($\eta =0.9$), the treated benefitted people will decrease when vaccine cost is cheaper (lower vaccine prices).

**Figure 11:**
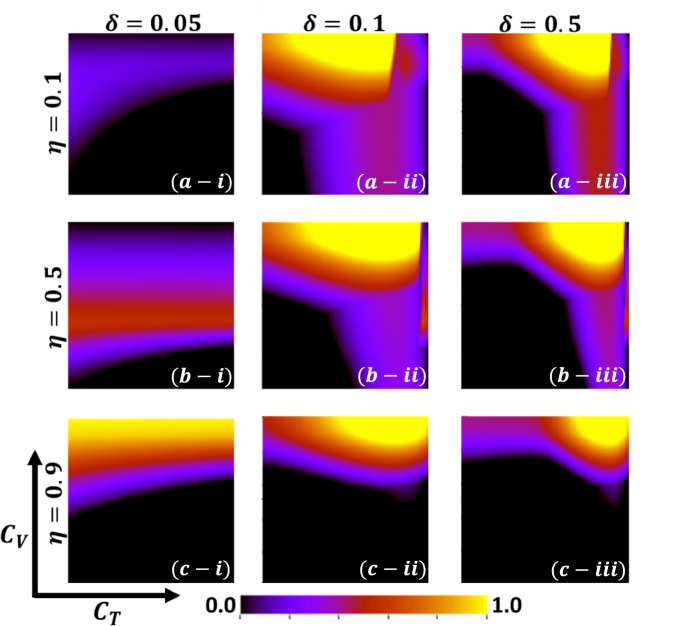
The 2D heatmapof the social efficient deficit (${SED}_{V}$) is present by varying two parameters: the *x*-axis contains treatment cost (${C}_{T}$) and the *y*-axis is vaccination cost (${C}_{V}$). In this figure, the first, second, and third rows display the result of varying the vaccine efficiency (a-*) $\eta =0.1$, (b-*) $\eta =0.5$, and (c-*) $\eta =0.9$. Also, the first, second and third columns show the result of varying the treatment duration rate: (*-i) $\delta =0.05$, (*-ii) $\delta =0.1$, and (*-iii) $\delta =0.5$. Other parameters are, $\beta =0.8333$, $\gamma =0.333$, and $\omega =0.01$.

**Figure 12:**
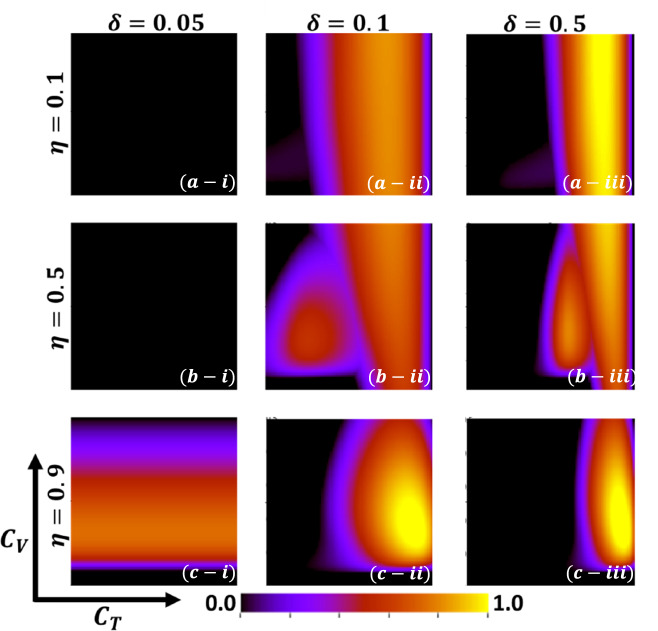
The 2D heatmap of the social efficient deficit (${SED}_{T}$) is present by varying two parameters: the *x*-axis contains treatment cost (${C}_{T}$) and the *y*-axis is vaccination cost (${C}_{V}$). In this figure, the first, second, and third rows display the result of varying the vaccine efficiency (a-*) $\eta =0.1$, (b-*) $\eta =0.5$, and (c-*) $\eta =0.9$. Also, the first, second and third columns show the result of varying the treatment duration rate: (*-i) $\delta =0.05$, (*-ii) $\delta =0.1$, and (*-iii) $\delta =0.5$. Other parameters are, $\beta =0.8333$, $\gamma =0.333$, and $\omega =0.01$.

In Figs. 11 and 12, we explore the idea of social efficiency deficit (SED) that explicitly reveals the underlying social dilemmas in the vaccination and treatment game. An individual's decision on taking provision (vaccine or treatment) that infers cooperation (C) or not accepting provision indicates defection (D) on the relative cost of vaccine and treatment. By exploring SED for vaccination and treatment, we generate the 2D heat map for ${SED}_{V}$ (Fig. 11) and ${SED}_{T}$ (Fig. 11) to visualize how SED varies as a function of ${C}_{V}$ and ${C}_{T}$. The region-colored black presented having no SED (no dilemma) in which society reached its stable situation, and the payoff at NE cannot be improved anymore. In Fig. 11, with a lower level of vaccination cost, ${SED}_{V}=0$, SED reaches its minimum point, meaning when vaccination cost is down, people will participate in the vaccine program (ALLC). Afterwards, it shows a monotonic increase when vaccine efficacy increases; the situation with a higher effective vaccine might inspire some people to participate vaccine program. Interestingly, we could also see the effect of treatment cost to arise dilemma situation. When $\eta \le 0.5$, comparatively lower reliability of vaccine, people somehow think about treatment provision. But, when the treatment cost is higher ${SED}_{V}$ is also arises, because people are trope in a dilemma situation. According to our findings, it can be concluded that we can minimize ${SED}_{V}$ by either lower the vaccine cost or improving the vaccine efficacy.

In Fig. 12a-i, b-i present completely no dilemma situation for $SED=0$. In the aspect of evolutionary game theory this no-dilemma situation is arises for $\delta <\gamma$. However, when vaccine effectiveness is high (panel c-i), few dilemmas observed that arises for middle cost of ${C}_{V}$ values. For comparatively higher and lower ${C}_{V}$, no dilemma situation is detected. We can conclude that when cost is lower people are participating vaccine program without any hesitation (ALLC) and for higher ${C}_{V}$, people are fully avoiding vaccination (ALLD). Meanwhile, when $\delta >\gamma$, dilemma situation arises for higher costly treatment; when ${C}_{T}>0$, we observed ${SED}_{T}>0$. Because, for higher cost of treatment people will think about to participate treatment or not that arises dilemma. On the other hand, for lower treatment cost people are fully cooperation about treatment and willingly to go for treatment (ALLC) that displayed no dilemma situation at all.

## Conclusion

This paper developed an SVITR epidemic model for the disease spread and the embedded vaccine and treatment behavioural dynamics by using extensive evolutionary game theory among the individuals in society. The most important contribution is that our new model gives an extensive framework that explains vaccination and treatment strategies considering different effectiveness, associated cost, and payoff structure on local time scales. Our model gives a clear context to quantify the social benefit and dilemmas entailed by vaccination and treatment games. Increasing the effectiveness of vaccination and lowering the vaccination cost increased the vaccination coverage, but that's how it reduced the final epidemic size. Lowering the treatment duration and improving treatment costs had a similar effect. Again, improving the vaccine efficacy and reducing treatment duration increased the treatment provision and average social payoff. Lowering vaccine efficacy and recovery rate, on the other hand, hampered treatment-seeking behaviour. Besides amplifying the voluntary vaccination game, our model introduced a new game expression with two directions: vaccination as a proactive measure and treatment as a retroactive measure. Thus, by applying proactive vaccination and retroactive treatment, we can investigate and understand the individuals' decisions regarding over-vaccination and perform proper strategies that reduce the divergency of infection and ensure the careful state of both antiviral treatment and vaccination. We also successfully introduced the concept of SBI to explore the idea of benefitted individuals when they are inclined to a specific strategy. Besides, our SED results also show the social dilemma situation for each strategy (vaccination or treatment). Finally, our results suggest that the ex-post treatment sometimes improves the final epidemic size that depends on other aspects, such as the reliability of vaccination and its cost, which recommend appropriate and careful treatment.

## Supplementary Information

Supplementary Information.

## Data Availability

The datasets generated and/or analyzed during the current study are not publicly available due to original code but are available from the corresponding author on reasonable request.
