# Supplementary material for: A cyclic behavioral modeling aspect to understand the effects of vaccination and treatment on epidemic transmission dynamics
Source: Sci Rep. 2023 May 23;13:8356. doi: 10.1038/s41598-023-35188-3 (PMC10205038; doi:10.1038/s41598-023-35188-3)
Supplement: Supplementary file 1 — Supplementary Information. [file 41598_2023_35188_MOESM1_ESM.pdf]

Supplementary Information for

A Cyclic behavioral Modeling aspect to understand the effects of Vaccination and Treatment on epidemic transmission dynamics

Abu Zobayer[1], Mohammad Sharif Ullah[2], K. M. Ariful Kabir[1]

Corresponding Author

K M Ariful Kabir, k.ariful@yahoo.com, km_ariful@math.buet.ac.bd.

This PDF file includes:

Supplementary text

Theoretical Analysis

Supporting Information Text and Theory

Mathematical Analysis

Here, we present some of the actual results related to the theoretical analysis of the model [56-61]. The primary goal is to get an asymptotic understanding of how the virus will propagate, ensuring that the model's explanations are accurate by requiring positivity and boundedness. We verify the asymptotic local stability study by finding the model's disease-free equilibrium. We also calculate the reproduction number and the existence of a uniformly stable situation for the exactness of the solution.

Model's positivity and boundedness of the solutions

In this part, we mainly focus on the proposed model's positivity and boundedness, which certifies the exactness of the model's solutions. Thus, for infected class $I(t)$, we may write,

$$I(t) \geq I^0 e^{-(\gamma+\tau)t}, \forall t \geq 0.$$

Similarly, the treatment class $T(t)$ is expressed as follows,

$$T(t) \geq R^0 e^{-\delta t}, \forall t \geq 0.$$

Furthermore, the recovered class $R(t)$ is,

$$R(t) \geq R^0 e^{-\omega t}, \forall t \geq 0.$$

Hence, the norm of the domain D_φ where $\varphi \in D_\varphi$ [61], put out in the following way

$$\|\varphi\|_\infty = \sup_{t \epsilon D_\varphi} |\varphi(t)|$$

Analogously, utilizing the overhead norm, the vaccinated and susceptible classes are likewise represented as follows,

$$\begin{aligned} \dot{V} &= xS - (1-\eta)\beta V I, \forall t \geq 0 \\ &\geq -\{(1-\eta)\beta I\}V, \forall t \geq 0 \\ &\geq -\{(1-\eta)\beta |I|\}V, \forall t \geq 0 \\ &\geq \{-(1-\eta)\beta \sup_{t \epsilon D_I} |I|\}V, \forall t \geq 0 \\ &\geq -\{(1-\eta)\beta \|I\|_\infty\}V, \forall t \geq 0 \end{aligned}$$

Therefore, $V(t) \geq V^0 e^{-\{(1-\eta)\beta\|I\|_\infty\}t}, \forall t \geq 0$.

In the same way, $S(t) \geq S^0 e^{-(\beta\|I\|_\infty + x)t}, \forall t \geq 0$.

Finally, we can conclude that the suggested model and its solution are both positive and bound.

Disease-free equilibrium (DFE) point and its stability

The disease-free equilibrium, symbolized by $\mathcal{E}_0$, is the point at which there is no infection in the population at the equilibrium stage, and all infected classes will have a zero value. To calculate the DFE point of the proposed model, we put $I = 0$ in the system (1-5). Then we get the DFE point of the current model is $\mathcal{E}_0 = (S^0, V^0, I^0, T^0, R^0) = (s^*, v^*, 0,0,0)$; $s^* + v^* = N(= 1)$.

Derivation of the basic reproduction (R_0) and effective reproduction number (R_e)

We calculate the basic reproduction number, R_0 to show stable equilibrium conditions to analyze the preliminary theoretical investigation. We consider the next-generation matrix [56] technique to evaluate the basic reproduction number as follows:

$$F = \begin{bmatrix} \beta s^* + \beta(1-\eta)v^* & 0 \\ 0 & 0 \end{bmatrix}$$

$$V = \begin{bmatrix} \gamma + \tau & 0 \\ -\tau & \delta \end{bmatrix}$$

$$FV^{-1} = \frac{1}{\delta(\gamma + \tau)} \begin{bmatrix} \delta[\beta s^* + \beta(1-\eta)v^*] & 0 \\ 0 & 0 \end{bmatrix}$$

As the basic reproduction number is the most considerable eigenvalue of FV^{-1} thus,

$$R_0 = \frac{\beta s^* + \beta(1-\eta)v^*}{\gamma + \tau}.$$

Determining an epidemiological model's effective reproduction number (R_e) is the same as the basic reproduction number [28-30]. The effective reproduction number can be estimated by multiplying the basic reproductive number and the proportion of the host population [68-71]. Therefore, the time-dependent reproduction number is known as the effective reproduction number R_e is,

$$R_e(t) = \frac{\beta}{\gamma + \tau} S(t) + \frac{\beta(1-\eta)}{\gamma + \tau} V(t).$$

Theorem 1. If $R_0 < 1$, then the disease-free equilibrium E_0 is locally asymptotically stable. If $R_0 > 1$, the disease-free equilibrium is unstable.

Proof: Let us compute the proposed model's Jacobian matrix is as

$$J = \begin{bmatrix} -\beta I - x & 0 & -\beta S & 0 & \omega \\ x & -(1-\eta)\beta I & -(1-\eta)\beta V & 0 & 0 \\ \beta I & (1-\eta)\beta I & \beta S + (1-\eta)\beta V - \gamma - \tau & 0 & 0 \\ 0 & 0 & \tau & -\delta & 0 \\ 0 & 0 & \gamma & \delta & -\omega \end{bmatrix}$$

Substituting the value of the DFE point E_0, we obtain

$$J(E_0) = \begin{bmatrix} -x & 0 & -\beta s^* & 0 & \omega \\ x & 0 & -(1-\eta)\beta v^* & 0 & 0 \\ 0 & 0 & \beta S^1 + \beta(1-\eta)v^* - \gamma - \tau & 0 & 0 \\ 0 & 0 & \tau & -\delta & 0 \\ 0 & 0 & \gamma & \delta & -\omega \end{bmatrix}$$

The characteristics equation $|J(E_0) - \lambda I| = 0$ has five roots, which are,

$$\lambda_1 = -\omega, \lambda_2 = -\delta, \lambda_3 = -x\,, \lambda_4 = -\gamma - \tau + \beta s^* + \beta(1-\eta)v^*,\ \ \lambda_5 = 0.$$

As all eigenvalues are negative or equal to zero, therefore, conferring to Routh-Hurwitz criteria [67], we can easily accomplish that the model is locally asymptotically stable at the disease-free equilibrium point E_0 whenever $R_0 < 1$ and unstable if $R_0 > 1$.

Existence of endemic equilibrium

In this part, we investigate the endemic equilibrium points denoted by $\mathcal{E}_* = (S^*, V^*, I^*, T^*, R^*)$ is whether it exists or not. For the endemic equilibrium point, we consider the equations as follows,

$$0 = -\beta SI - xS + \omega R$$

$$0 = xS - (1-\eta)\beta VI$$

$$0 = \beta SI + (1-\eta)\beta VI - (\gamma+\tau)I$$

$$0 = \tau I - \delta T$$

$$0 = \delta T + \gamma I - \omega R.$$

After some simplification, we obtain,

$$S^* = \frac{\gamma+\tau}{\beta I^* + x} I^*,$$

$$V^* = \frac{x(\gamma+\tau)}{\beta(\beta I^* + x)(1-\eta)},$$

$$T^* = \frac{\tau}{\delta} I^*,$$

$$R^* = \frac{\gamma+\tau}{\omega} I^*.$$

Theorem 4. The endemic equilibrium point E^* is locally asymptotically stable and unstable whenever $R_0 > 1$.

Proof: Remember that the system's (1.1-1.6) Jacobian exists at any point of equilibrium (S, M, V, E, I, R), and we obtain

$$J = \begin{bmatrix} -\beta I - x & 0 & -\beta S^* & 0 & \omega \\ x & -(1-\eta)\beta I & -(1-\eta)\beta V & 0 & 0 \\ \beta I & (1-\eta)\beta I & \beta S + (1-\eta)\beta V - \gamma - \tau & 0 & 0 \\ 0 & 0 & \tau & -\delta & 0 \\ 0 & 0 & \gamma & \delta & -\omega \end{bmatrix}$$

Hence, at the endemic equilibrium point E_1^*, the desired Jacobian matrix is

$$J(E^*) = \begin{bmatrix} -\beta I^* - x & 0 & -\beta S^* & 0 & \omega \\ x & -(1-\eta)\beta I^* & -(1-\eta)\beta V^* & 0 & 0 \\ \beta I^* & (1-\eta)\beta I^* & \beta S^* + (1-\eta)\beta V^* - \gamma - \tau & 0 & 0 \\ 0 & 0 & \tau & -\delta & 0 \\ 0 & 0 & \gamma & \delta & -\omega \end{bmatrix}$$

The roots of the characteristic equations $|J(E_1^* - \lambda I_6|$ satisfies the following equation:

$$\lambda^5 + a_4\lambda^4 + a_3\lambda^3 + a_2\lambda^2 + a_1\lambda + a_0 = 0, \quad \text{(A1)}$$

where,

$$a_4 = -A_{11} + A_{13} - A_{16} + \delta + \omega,$$

$$a_3 = -A_{11}A_{13} + A_{13}A_{14} - A_{12}A_{15} + A_{11}A_{16} - A_{13}f - a\delta + A_{13}\delta - f\delta - a\omega + A_{13}\omega - A_{16}\omega + \delta\omega,$$

$$a_2 = -A_{11}A_{13}A_{14} - A_{12}A_{13}A_{15} + A_{11}A_{13}A_{16} - A_{12}A_{13}x - A_{11}A_{13}\delta + A_{13}A_{14}\delta - A_{12}A_{15}\delta + A_{11}A_{16}\delta - A_{13}A_{16}\delta - A_{11}A_{13}\omega + A_{13}A_{14}\omega - A_{12}A_{15}\omega + A_{11}A_{16}\omega - A_{13}A_{16}\omega - A_{15}\gamma\omega - A_{11}\delta\omega + A_{13}\delta\omega - A_{16}\delta\omega,$$

$$a_1 = -A_{11}A_{13}A_{14}\delta - A_{12}A_{13}A_{15}\delta + A_{11}A_{13}A_{16}\delta - A_{12}A_{13}x\delta - A_{12}A_{13}e\omega + A_{11}A_{13}A_{16}\omega - A_{12}A_{13}x\omega - A_{13}A_{15}\gamma\omega - A_{13}x\gamma\omega - A_{11}A_{13}\delta\omega + A_{13}A_{14}\delta\omega - A_{12}A_{15}\delta\omega + A_{11}A_{16}\delta\omega - A_{13}A_{16}\delta\omega - A_{15}\gamma\delta\omega - A_{15}\delta\tau\omega - A_{11}A_{13}A_{14}\omega,$$

$$a_0 = -A_{13}(A_{11}A_{14}\delta\omega + A_{12}A_{15}\delta\omega - A_{11}A_{16}\delta\omega + A_{12}x\delta\omega + A_{15}\gamma\delta\omega + x\gamma\delta\omega + A_{15}\delta\tau\omega + x\delta\tau\omega),$$

$$A_{11} = -\beta I^* - x,$$

$$A_{12} = -\beta S^*,$$

$$A_{13} = (1-\eta)\beta I^*,$$

$$A_{14} = (1-\eta)\beta V^*,$$

$$A_{15} = \beta I^*,$$

$$A_{16} = \beta S^* + (1-\eta)\beta V^* - \gamma - \tau.$$

It is simple to demonstrate that all of the roots of equation (A1) will have a negative real portion if $R_0 > 1$ and that the coefficients of equation (A1) will fulfill the Routh-Hurwitz condition [67]. The endemic equilibrium point will thus be locally asymptotically stable for $R_0 > 1$.

Theorem 2. Let the Lyapunov function L_f for the endemic equilibrium point $\mathcal{E}_*$ is $\{S, V, I, T, R\}, L_f < 0$. We have to prove that $\mathcal{E}_*$ is globally asymptotically stable for $R_0 > 1$.

Proof: Let us suppose that the Lyapunov function is,

$$L_f(S,V,I,T,R) = \left(S - S^* - S^* log\frac{S^*}{S}\right) + \left(V - V^* - V^* log\frac{V^*}{V}\right) + \left(I - I^* - I^* log\frac{I^*}{I}\right) + \left(T - T^* - T^* log\frac{T^*}{T}\right) + \left(R - R^* - R^* log\frac{R^*}{R}\right) \quad \text{(A2)}$$

After applying the first derivative on both sides of the equation (A2) concerning t, one can obtain

$$\dot{L_f} = \left(\frac{S-S^*}{S}\right)\dot{S} + \left(\frac{V-V^*}{V}\right)\dot{V} + \left(\frac{I-I^*}{I}\right)\dot{I} + \left(\frac{T-T^*}{T}\right)\dot{T} + \left(\frac{R-R^*}{R}\right)\dot{R} \quad \text{(A3)}$$

Substituting the values of $\dot{S}, \dot{V}, \dot{I}, \dot{T}, \dot{R}$ from equation (1-5) in equation (A3), we get,

$$\dot{L_f} = \left(\frac{S-S^*}{S}\right)\{-\beta SI - xS + \omega R\} + \left(\frac{V-V^*}{V}\right)(xS - (1-\eta)\beta VI) + \left(\frac{I-I^*}{I}\right)(\beta SI + (1-\eta)\beta VI - (\gamma+\tau)I) + \left(\frac{T-T^*}{T}\right)(\tau I - \delta T) + \left(\frac{R-R^*}{R}\right)(\delta T + \gamma I - \omega R) \quad \text{(A4)}$$

Substitute $S = S - S^*, V = V - V^*, I = I - I^*, T = T - T^*, R = R - R^*$ in equation (A4), one can obtain

$$\dot{L_f} = \dot{L_f} = \left(\frac{S-S^*}{S}\right)\{-\beta(S-S^*)(I-I^*) - x(S-S^*) + \omega(R-R^*)\} + \left(\frac{V-V^*}{V}\right)\{x(S-S^*) - (1-\eta)\beta(V-V^*)(I-I^*)\} + \left(\frac{I-I^*}{I}\right)\{\beta(S-S^*)(I-I^*) + (1-\eta)\beta(V-V^*)(I-I^*) - (\gamma+\tau)(I-I^*)\} + \left(\frac{T-T^*}{T}\right)\{\tau(I-I^*) - \delta(T-T^*)\} + \left(\frac{R-R^*}{R}\right)\{\delta(T-T^*) + \gamma(I-I^*) - \omega(R-R^*)\} \quad \text{(A5)}$$

Equation (A5) can be written as

$$\dot{L_f} = \psi_1 - \psi_2 \quad \text{(A6)}$$

Where,

$$\psi_1 = \omega\frac{S-S^*}{S}R + \beta\frac{(S-S^*)^2}{S}I^* + x\frac{V-V^*}{V}S + (1-\eta)\beta\frac{(V-V^*)^2}{V}I^* + \beta\frac{(I-I^*)^2}{I}S + (1-\eta)\beta\frac{(I-I^*)^2}{I}V + \tau\frac{T-T^*}{T}I + \delta\frac{R-R^*}{R}T + \gamma\frac{R-R^*}{R}I$$

and

$$\psi_2 = \beta\frac{(S-S^*)^2}{S}I + x\frac{(S-S^*)^2}{S} + \omega\frac{S-S^*}{S}R^* + x\frac{V-V^*}{V}S^* + (1-\eta)\beta\frac{(V-V^*)^2}{V}I + \beta\frac{(I-I^*)^2}{I}S^* + (1-\eta)\beta\frac{(I-I^*)^2}{I}V^* + (\gamma+\tau)\frac{(I-I^*)^2}{I} + \tau\frac{T-T^*}{T}I^* + \delta\frac{(T-T^*)^2}{T} + \delta\frac{R-R^*}{R}T^* + \gamma\frac{R-R^*}{R}I^* + \omega\frac{(R-R^*)^2}{R}.$$

It is evident that $\dot{L_f} < 0$ when $\psi_1 < \psi_2$.

Therefore, for $S = S^*, V = V^*, I = I^*, T = T^*, R = R^*$

From equation (14),$\Rightarrow 0 = \psi_1 - \psi_2$, Implies$\Rightarrow \dot{L_f} = 0$. (A7)

In that case, according to Lasalle's invariance principle, the endemic equilibrium point $\mathcal{E}_*$ is globally asymptotically stable in Γ when $\psi_1 < \psi_2$ for the compact invariant set,

$$\left\{(S^*, V^*, I^*, R^*) \in \Gamma : \dot{L_f} = 0\right\} \quad \text{(A8)}$$

Existence of a uniformly stable solution

To establish the exactness of a uniformly stable solution, assume that

$$\dot{S} = -\beta SI - xS + \omega R = f_1(S, V, I, T, R)$$

$$\dot{V} = xS - (1-\eta)\beta VI = f_2(S, V, I, T, R)$$

$$\dot{I} = \beta SI + (1-\eta)\beta VI - (\gamma+\tau)I = f_3(S, V, I, T, R)$$

$$\dot{T} = \tau I - \delta T = f_4(S, V, I, T, R)$$

$$\dot{R} = \delta T + \gamma I - \omega R = f_5(S, V, I, T, R).$$

For the total population $N(t)(= 1)$, we may write,

$\Pi = \{(S(t) + V(t) + I(t) + T(t) + R(t)) \in R^5 : |\zeta(i)| \leq N(t)$ and $t \in [0, T(time\ period)]$.

Thus, over Π, we have

$$\frac{\partial f_1}{\partial S} = -\beta I - x \Rightarrow \left|\frac{\partial f_1}{\partial S}\right| \leq a_{11}; \frac{\partial f_1}{\partial V} = 0 = f_1(V) = a_{12};$$

$$\frac{\partial f_1}{\partial I} = -\beta S \Rightarrow \left|\frac{\partial f_1}{\partial I}\right| \leq a_{13}; \frac{\partial f_1}{\partial T} = 0 = f_1(T) = a_{14}; \frac{\partial f_1}{\partial R} = \omega \Rightarrow \left|\frac{\partial f_1}{\partial R}\right| \leq a_{15};$$

$$\frac{\partial f_2}{\partial S} = x \Rightarrow \left|\frac{\partial f_2}{\partial S}\right| \leq a_{21}; \frac{\partial f_2}{\partial V} = -(1-\eta)\beta I \Rightarrow \left|\frac{\partial f_2}{\partial V}\right| \leq a_{22};$$

$$\frac{\partial f_2}{\partial I} = -(1-\eta)\beta V \Rightarrow \left|\frac{\partial f_2}{\partial I}\right| \leq a_{23}; \frac{\partial f_2}{\partial T} = 0 = f_2(T) = a_{24}; \frac{\partial f_2}{\partial R} = 0 = f_2(R) = a_{25};$$

$$\frac{\partial f_3}{\partial S} = \beta I \Rightarrow \left|\frac{\partial f_3}{\partial S}\right| \leq a_{31}; \frac{\partial f_3}{\partial V} = (1-\eta)\beta I \Rightarrow \left|\frac{\partial f_3}{\partial V}\right| \leq a_{32};$$

$$\frac{\partial f_3}{\partial I} = \beta S + (1-\eta)\beta V - (\gamma + \tau) \Rightarrow \left|\frac{\partial f_3}{\partial I}\right| \leq a_{33}; \frac{\partial f_3}{\partial T} = 0 = f_3(T) = a_{34}; \frac{\partial f_3}{\partial R} = 0 = f_3(R) = a_{35};$$

$$\frac{\partial f_4}{\partial S} = 0 = f_4(S) = a_{41}; \frac{\partial f_4}{\partial V} = 0 = f_4(V) = a_{42}; \frac{\partial f_4}{\partial I} = \tau \Rightarrow \left|\frac{\partial f_4}{\partial I}\right| \leq a_{43};$$

$$\frac{\partial f_4}{\partial T} = -\delta \Rightarrow \left|\frac{\partial f_4}{\partial T}\right| \leq a_{44}; \frac{\partial f_4}{\partial R} = 0 = f_4(R) = a_{45};$$

$$\frac{\partial f_5}{\partial S} = 0 = f_5(S) = a_{51}; \frac{\partial f_5}{\partial V} = 0 = f_5(V) = a_{52}; \frac{\partial f_5}{\partial I} = \gamma \Rightarrow \left|\frac{\partial f_5}{\partial I}\right| \leq a_{53};$$

$$\frac{\partial f_5}{\partial T} = \delta \Rightarrow \left|\frac{\partial f_5}{\partial T}\right| \leq a_{54}; \frac{\partial f_5}{\partial R} = -\omega \Rightarrow \left|\frac{\partial f_5}{\partial R}\right| \leq a_{55};$$

Here, the constants $a_{ij}(i \geq 1$ and $j \leq 5)$ all are positive. Therefore, the suggested model's five functions, namely $f_1, f_2, \cdots, f_5$ all are satisfied well-known Lipchitz condition [62-66].

Strength number

We use the suitable strength numbers approach to determine the waving tendency in proposed epidemic dynamics [61]. First, we determined the recommended model's strength number under the assumption of a limited population, N, by analyzing the partial first derivative of the infected class using next-generation matrix techniques as follows:

$$\beta SI = \frac{\beta SI}{N}, (1-\eta)\beta VI = (1-\eta)\beta\frac{VI}{N}.$$

Therefore,

$$\frac{\partial^2}{\partial I^2}\left[\beta\frac{SI}{N} + (1-\eta)\beta\frac{VI}{N}\right] = \beta S\frac{\partial}{\partial I}\left(\frac{N - \dot{N}I}{N^2}\right) + (1-\eta)\beta V\frac{\partial}{\partial I}\left(\frac{N - \dot{N}I}{N^2}\right)$$

$$= -\beta\frac{S}{N^2} - (1-\eta)\beta\frac{V}{N^2}.$$

Then,

$$F = \begin{bmatrix} -\frac{\beta}{N^2} - \frac{(1-\eta)\beta}{N^2} & 0 \\ 0 & 0 \end{bmatrix}$$

and

$$FV^{-1} = \frac{1}{\delta(\gamma+\tau)} \begin{bmatrix} \delta\left[-\frac{\beta}{N^2} - \frac{(1-\eta)\beta}{N^2}\right] & 0 \\ 0 & 0 \end{bmatrix}.$$

Therefore, as previously, from the spectral radius of $\rho(FV^{-1})$ for defining the epidemic wave, the desired strength number is denoted by R_{SN},

$$R_{SN} = -\frac{\beta+(1-\eta)\beta}{N^2(\gamma+\tau)} \tag{A9}$$

When $R_{SN} \leq 0$, the disease can only produce one wave, and the infection class would quickly drop below or equal to the equilibrium of disease-free conditions. However, when $R_{SN} \geq 0$, multi-waving scenarios are revealed. Here, all parameters of the suggested model are well-defined and positive. More precisely, $\beta, \gamma, \tau \geq 0,$ and $0 \leq \eta \leq 1$, which shows that the proposed model's strength number $R_{SN} \leq 0$ represents only one wave.

Geometrical interpretation of Strength Number

The second-order derivative usually depicts the concavity or curvature of any graph. In epidemic models, a concept like this from fundamental calculus is routinely applied to observe the situation of several layers or waves of epidemic disease cases. To illustrate the second-order time derivative study of our suggested model, we exemplify it below as follows:

$$\ddot{S} = -\beta\dot{S}I - \beta S\dot{I} - x\dot{S} + \omega\dot{R},$$

$$\ddot{V} = x\dot{S} - (1-\eta)\beta\dot{V}I - (1-\eta)\beta V\dot{I},$$

$$\ddot{I} = \beta\dot{S}I + \beta S\dot{I} + (1-\eta)\beta\dot{V}I + (1-\eta)\beta V\dot{I} - (\gamma+\tau)\dot{I},$$

$$\ddot{T} = \tau\dot{I} - \delta\dot{T},$$

$$\ddot{R} = \delta\dot{T} + \gamma\dot{I} - \omega\dot{R}. \tag{A10}$$

Putting the value of the first derivative $\dot{S}, \dot{V}, \dot{I}, \dot{T}, \dot{R}$ from equation (1-5) in equation (A9), we get,

$$\ddot{I} = \beta(-\beta SI - xS + \omega R)I + (1-\eta)\beta\{xS - (1-\eta)\beta VI\}I + \{\beta S + (1-\eta)\beta V - (\gamma+\tau)\}\{\beta SI + (1-\eta)\beta VI - (\gamma+\tau)I\},$$

$$\ddot{T} = \tau\{(\beta SI + (1-\eta)\beta VI - (\gamma+\tau)I)\} - \delta(\tau I - \delta T). \tag{A11}$$

By using the disease-free equilibrium point, we can demonstrate the concavity of the system of nonlinear ODEs (equation A11). The inflection point occurs when the time derivative of the second order equals zero. Concave up arises if it is more significant than zero, and concaves down if it is less meaningful than zero. Using the system equation (A11) and the disease-free equilibrium point $\mathcal{E}_0$, we may conclude that $\mathcal{E}_0$ cannot be concave up or down,

$$\ddot{I} = 0,$$

$$\ddot{T} = 0. \tag{A12}$$

Equation (A11) shows that for all second-order time derivatives utilized in the computation of concavity, we only have the case for the inflection or stationary points. In conclusion, the model equation (A11) only provides the infection or the fixed points for the second-order model equation (A10) at the disease-free equilibrium points $\mathcal{E}_0$ instead of the concave up and concave down.
